# Human phosphoglycerate dehydrogenase produces the oncometabolite D-2-hydroxyglutarate and promotes histone methylation

**DOI:** 10.1186/2049-3002-2-S1-P75

**Published:** 2014-05-28

**Authors:** Jing Fan, Xin Teng, Ling Liu, Ryan Looper, Joshua Rabinowitz

**Affiliations:** 1 Lewis-Sigler Institute for Integrative Genomics and Department of Chemistry, Princeton University, Princeton, NJ, USA; 2 Department of Chemistry, University of Utah, Salt Lake City, UT, USA; 3 Rutgers Cancer Institute of New Jersey, New Brunswick, NJ, USA

## Background

Human phosphoglycerate dehydrogenase (PHGDH), the first enzyme in the serine biosynthetic pathway, is genomically amplified in various tumors [1-3]. In such cells, PHGDH knockdown is not fully rescued by exogenous serine [2], suggesting possible additional roles for the enzyme.

## Materials and methods

To analyze the catalytic activity of PHGDH on possible alternative substrates, we conducted *in vitro* biochemical assays on recombinant human PHGDH. The reaction rate in the reductive direction with various substrates was monitored based on NADH consumption, measured by absorbance at 340 nm. The identity and chirality of products were analyzed by liquid chromatography-mass spectrometry (LC-MS) and by gas chromatography-mass spectrometry (GC-MS) after (R)-2-butanol derivatization. To analyze the intracellular function of PHGDH, we generated stable PHGDH knockdown cell lines, using two shRNA sequences. Metabolite levels in the knockdown cells were analyzed by LC-MS [4], and DNA and histone methylation was analyzed by immunoblotting.

## Results

Here we show that, in addition to catalyzing oxidation of 3-phosphoglycerate, PHGDH catalyzes NADH-dependent reduction of α-ketoglutarate to the oncometabolite D-2-hydroxyglutarate (D-2HG) and promotes histone methylation. The impact of PHGDH knockdown was studied in three cell lines with amplified PHGDH: MDA-MB-468, BT-20, and HCC70. Knockdown of PHGDH decreased 2HG levels in MDA-MB-468 and BT-20 cells, but not HCC70 cells (which had the highest 2HG levels of the three tested cell lines, with levels in MDA-MB-468 and BT-20 cells far below those found in cells with mutant IDH). These results suggest that PHGDH contributes to physiological 2HG pools, but may not produce high enough concentrations to cause pathology. Interestingly, in all three cell lines, PHGDH knockdown substantially decreased histone methylation. The mechanism underlying the decreased methylation upon PHGDH knockdown remains unclear, but it could be restored in MDA-MB-468 and BT-20 cells by addition of D-2HG ester.

## Conclusion

PHGDH may promote histone methylation by mechanisms including 2-HG production.
